# Fractal Kinetic Analysis of Biomass Hydrothermal Carbonization

**DOI:** 10.1021/acsomega.5c03676

**Published:** 2025-07-30

**Authors:** Alberto Gallifuoco, Luca Taglieri

**Affiliations:** Department of Industrial and Information Engineering & Economics, 90345 University of L’Aquila, Piazzale Ernesto Pontieri 1, Monteluco di Roio, 67100 L’Aquila, Italy

## Abstract

This paper introduces fractal analysis to study the kinetics of biomass hydrothermal carbonization. The reacting water-biomass slurry is a complex system leading to hydrochar by microscale reactions constrained into fractal topological boundaries. Literature and purpose experimental data check equations adapted from the fractal-like repertoire. More general models are derived from a shortcut, stochastic-based formalism, avoiding the mathematical sophistication of fractal calculus. Fractal equations explain observed data better and with fewer parameters than traditional mass-action network models (0.91202 < *R* ^2^ < 0.99998, average 0.97957) over a wide range of biomass and operational conditions. Exploratory experiments highlight the surface fractal dimension of hydrocars and their variation concerning that of parent biomasses (from 2.00 to 2.84). The confluence of fitting success and evidence of HC fractality encourages prosecuting research for making fractal kinetics a fully fledged tool of hydrothermal carbonization studies.

## Graphical abstract

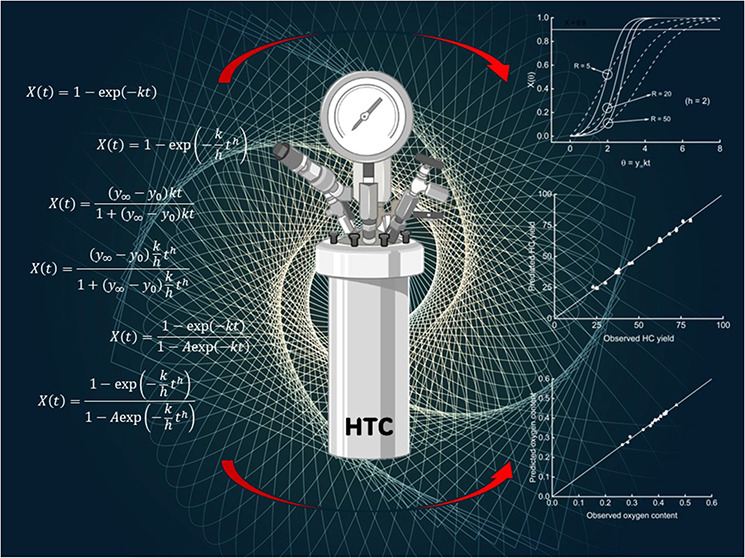

## 1 Introduction

The precepts of waste minimization and circular economy oblige chemical engineers to develop new sustainable schemes for exploiting industrial residues thoroughly. Process innovation, intensification, and integration under environmental safety constraints challenge the design of waste conversions simultaneously oriented to energy and value-added products.^1,2^ Countries with established agroforestry vocations actively study how to make available the vast energy and chemical sources confined in the residual lignocellulosic biomass.^3,4^ Whatever the selected route, the correct implementation at the industrial scale relies on a solid knowledge of the process rate. Among the possible pathways, keen attention focuses on hydrothermal carbonization (HTC), a thermal process that is nowadays broadly studied,^5^ resulting in significant patenting.^6^ HTC’s versatility is handy for treating high-moisture agro-industrial feedstocks, intrinsically seasonal and variable, under mild conditions without the pretreatment costs required by other thermal conversions.^7^ During HTC, biomass slurry and hot, compressed subcritical water yield many different reactions. The final products are the hydrochar (HC), an energy-densified solid, and the bio-oil, or process water (PW), an aqueous phase hosting many different dissolved molecules, potentially exploitable as chemical building blocks. The side-product, a lesser quantity of gas, prevalently CO_2_, is disregarded. This concise description accounts for HTC’s centrality in designing industrial plants for the simultaneous conversion of waste biomass to energy and value-added products. State-of-the-art chemical reaction engineering emerges from many well-assessed literature reviews. This contribution’s selected references are a sufficiently exhaustive guide to kinetic models, the different techniques, and the evidence of their experimental validation.^8–10^ The attentive perusal of such kinds of studies leaves the reader with the perception that modeling remains an open challenge for bringing knowledge of HTC to a completeness. The underlying reason is the hardship of managing the inherent complexity due to many-body interactions occurring during the process. Reducing the kinetics into a few first-order Arrhenius-type steps works well for the HTC of pure carbohydrates.^11^ Extending the approach to heterogeneous biomasses requires a laborious and skillful use of lumping techniques. Successful examples exist, although rarer.^12–14^ These models, based on the homogeneity of the reaction environment and spatial distribution of reactants, explain the experimental data satisfactorily. Still, typically, the fittings return some noninteger order of reaction. The outcome, not undermining the approach’s accuracy, suggests that the rate observed in the macro-environment depends on other factors besides the bulk-phase concentrations.

The authors believe that the solid matrix microscale geometry could play a role. Such property is not embeddable into traditional kinetic models. Although process models recently gave valiant advancements through computational techniques,^15^ the inclusion of geometrical information is still unrealistic. Fortunately, the researchers begin highlighting the dynamics of structural changes during the solid transformation from raw biomass to HC,^16^ and the experimental evidence needed for triggering the appearance of innovative models is proliferating. Geometry should be raised to the status of an additional variable to monitor alongside bulk properties, e.g., carbon and energy content, to describe HC formation accurately. Chemical engineering is now scaling down to nanodimensions,^17^ which could culturally nurture the necessary change in perspective. This contribution suggests a possible way to investigate the relationship between the microscale three-dimensional (3D) organization of the solid matrix and the observed kinetics. HTC belongs to heterogeneous, liquid–solid reactions and mainly entangles cellulose and hemicellulose. Lignin, the third component of lignocellulosic biomass, is substantially inert unless one pushes the severity of the process toward the limits of the hydrothermal liquefaction. Roughly speaking, lignin is a proto-hydrochar already contained in the raw material. Literature reports robust evidence on the fractal geometry of the lignin structure.^18–21^ Fractality also appears in artificial carbonaceous materials like nanotube networks^22^ and pyrolytic biochar.^23^ Also, the idea of treating the reacting biomass as a complex system appears.^24,25^ In this way, considering HC formation as the result of microscale reactions constrained into topological boundaries is worth investigating. The fibers of reacting cellulose and hemicellulose join tightly to the lignin matrix so that the fractality of this latter could reverberate in the HTC pattern.

To the authors’ knowledge, this contribution is the first example of fractal techniques applied to HTC kinetics studies. The task of this paper is twofold: to show how suitable HTC’s overall kinetic equations could originate from fractality and to assess models against experimental evidence from HTC of biomasses with different lignin amounts. The test data set comes from the literature and on-purpose experiments (three raw lignocellulosic waste biomasses, FIR, POTATO, and CARROT). These latter materials are also the object of preliminary tests for determining fractal dimension.

## 2 Modeling

HC formation is an inherently autocatalytic process. Initially, the hydrolytic detachment of oligomers from cellulose and hemicellulose refines the raw biomass, producing the so-called “primary” hydrochar. The hydrolytic process exposes a progressively increasing number of sites to water action. Dissolved substances accumulate in the PW and speed up condensation reactions responsible for producing carbonaceous nanoparticles, which join the solid matrix, increasing carbon content. This latter process becomes significant at longer reaction times, and the resulting hydrochar is “secondary”. As time elapses, macromolecular substrate shortage depletes the detachment rate, and the consumption of the dissolved molecules diminishes that of the condensation reactions. Consequently, the overall transformation rate from biomass to HC increases and passes to a maximum, progressively slowing down. The behavior appears even when the starting materials are pure carbohydrates or monosaccharides. In these cases, the initial rate acceleration depends mainly on the accumulation of acetic acid in the PW, which acts catalytically, as hydrolysis reactions are promoted in a low-pH environment. Long-time substrate depletion slows down progressively. Whatever the approach, kinetic modeling should keep in mind the above scenario.

Let *y*(*t*) be any time-dependent property signaling the reaction advancing from the initial value *y*_0_, i.e., due to the pre-existing raw biomass, and the final value (*y*_∞_). The simplest mass-action rate equation, which complies with the required dynamics, is

1 $$\frac{dy}{dt}=\mathrm{ky}\left(y_{\infty }-y\right)$$

The only kinetic parameter of eq 1 is *k*, which steers the intrinsic frequency of the reaction. Upon integration, one gets

2 $$y\left(t\right)=y_{\infty }\frac{1}{1+\frac{y_{\infty }-y_{0}}{y_{0}}\exp\left(-y_{\infty }\mathrm{kt}\right)}$$

Kopelman^26^ showed in a fascinating pioneer paper that elementary reactions could lead to power-law kinetics in the presence of diffusion limits or dimensional restrictions or when occurring on fractal surfaces. Neither eq 2 nor those deriving from more complex, multistep models could show power-law behavior. Fractal geometry first entered reaction kinetics in the above reference by introducing time-dependent rate coefficients and variable reaction orders into mass-action rate equations. Fractalizing time, eq 1 becomes

3 $$\frac{dy}{dt}=\frac{k}{t^{1-h}}y\left(y_{\infty }-y\right)$$

The new parameter, *h*, brings to the fractal-like kinetics and modulates the deviation from the traditional mass-action rate equation. Integration of eq 3 gives

4 $$y\left(t\right)=y_{\infty }\frac{1}{1+\frac{y_{\infty }-y_{0}}{y_{0}}\exp\left(-\frac{y_{\infty }k}{h}t^{h}\right)}$$

The fractal-like modeling approach modifies well-established equations by making the kinetic constants time-dependent. The postvalidation of the technique leverages the improvement of fittings. Accordingly, eqs 2 and 4 are worth considering for the entry-level assessment of the fractal origin of HTC experimental data. More models are necessary for an exhaustive analysis; the present study shows a way to get them.

Equations already assessed in other fields could model HTC by analogy. The literature presents a broad spectrum of fractal-like applications to phenomena occurring in the presence of carbonaceous materials. Examples are the kinetics of liquid-phase adsorption,^27–29^ bioprocesses,^30,31^ and catalysis.^32^

Adsorption is a fertile field for mining equations since diffusional resistance and geometrical hindrance steering the interaction between the sorbent and solution could establish the prerequisite of system complexity.^33^ In fractal-like adsorption kinetics, the microstructural heterogeneity of the solid matrix induces a distribution of molecule transport rates and adsorptive energetic interactions. The corresponding macroscopic symptom is that equations containing time-dependent kinetic and equilibrium constants work well. Analogous phenomena could occur between HC and PW.

Mass transfer resistance (water from bulk to pores and hydrolysis products from the matrix to the bulk) could affect the observed reaction rate. Fractal media generate diffusion coefficient distributions, and this specificity has repercussions in the time-variable kinetic constants. Fractality could also steer the secondary process of hydrochar accretion. The pre-existing matrix acts as a nucleation agent for the carbon nanoparticles, transferring from the bulk phase to the solid. A schematic mechanistic diagram appears in the Supporting Information to illustrate the basic feature of the previous scenario.

A further set of more sophisticated experiments is conceivable for reinforcing the analogy between adsorption and reaction. Plenty of adsorption kinetic equations could also apply to HTC. However, excessive proliferation is unnecessary for the introductory scope of this study. The discussion section makes use of a focused selection of referenced equations.

Although fractal-like equations furnish efficient information for setting up mass balances, the design of an industrial HTC reactor would benefit from models obtained by a formalism, starting from a universal parent function. This more ambitious goal, not *a priori* guaranteed of success, would repay the effort with a solid foundation for kinetic models and a clear physical meaning of their parameters. The remaining part of this section deals with a possible way to obtain the result.

A vast repertoire of studies supports fractal kinetic analysis^34^ and mathematical tools for handling differential equations relevant to chemical engineering applications. An example of these techniques is fractal calculus, which has existed for a long time.^35^ A shortcut for obtaining physically meaningful kinetic equations while avoiding too complex mathematical sophistication appeared during the last two decades,^36,37^ giving rise to fruitful follow-ups. An adaptation to the new field of HTC could go as follows:

Let us consider a generalization of eq 3:

5 $$\frac{dy}{dt}=f\left(t\right)y\left(1-y\right)$$

The solution of this Bernoulli’s differential equation depends upon the choice of the function *f*(*t*), which depends on the kinetic phenomenon and the environment in which it occurs and steers the intrinsic rate of the HTC process. The dynamics described by eq 5 belong to the Markov birth-and-death processes. Remarkably, Markov processes entered HTC modeling as a statistics-based mechanistic interpretation in a previous paper,^38^ which considered the HTC time course as a cumulative distribution function (CDF) of characteristic reaction times. Since fractal kinetics management via distribution functions has found convincing examples in suggestive trailblazer papers,^39,40^ the extension to HTC is worth considering.

Setting *f*(*t*) = 1 eq 5 returns the solution eq 2, which is the well-known Verhulst logistic equation. More in general, eq 5 generates the CDFs belonging to the Burr family,^41^ a set of distributions extensively used in applied science. The most recurrent equation, the Burr XII, found a recent application to HTC using the Shannon entropy maximization.^42^ The Burr XII appears naturally as a reference distribution in modeling the kinetics of complex systems and, in the authors’ opinion, should be a full member of the toolset of HTC researchers. To obtain the Burr XII from eq 5, one could set for the fractal forcing function the three-parameter equation:

6 $$f\left(t\right)=\frac{1}{t}\frac{a{\left(\frac{t}{\tau }\right)}^{a}}{(1+h{\left(\frac{t}{\tau }\right)}^{a})\left[1-{\left(1+h{\left(\frac{t}{\tau }\right)}^{a}\right)}^{-1/h}\right]}$$

The meaning of the parameters is as follows: *h* is the fractional order of reaction, *a* modulates the rate constant variation in time, and τ is the characteristic time. Upon insertion of eq 6 in eq 5, one gets the general solution.

7 $$y\left(t\right)=y_{\infty }-\left(y_{\infty }-y_{0}\right)\left[\frac{1}{{\left(1+h{\left(\frac{t}{\tau }\right)}^{a}\right)}^{1/h}}\right]$$

Solution 7 contains the end-point value *y*_∞,_ so it describes the entire evolution of the property *y* from the initial value, the raw biomass, to the final one, the ultimate HC. The medium temperature HTC processes come to a halt within a few hours, usually 2–3. More prolonged retention in the reactor would not reward significant amelioration of the final product since lignin refinement, excluded by this study, becomes appreciable only at higher temperatures. An experimental measure on the raw biomass could give *y*_0_. The property detected in the end-point HC (*y*_f_) is a reliable estimate of *y*_∞_. eq 7 is, therefore, a versatile, three-parameter model for fitting experimental data. Subtracting *y*_0_ from both sides of eq 7 and dividing by (*y*_0_ – *y*_∞_) one gets

8 $$\frac{y\left(t\right)-y_{0}}{y_{\infty }-y_{0}}=X\left(t\right)=\frac{{\left(1+h{\left(\frac{t}{\tau }\right)}^{a}\right)}^{1/h}-1}{{\left(1+h{\left(\frac{t}{\tau }\right)}^{a}\right)}^{1/h}}$$

­(*X*) is the ratio of the instantaneous variation to the end-point one and, spanning from 0 to 1, covers the time evolution of both increasing and decreasing quantities. In eq 8, when the parameters *a* and *h* have certain specific values, the fractal functionality yields well-known equations widely used in reaction and adsorption kinetic studies.^36^ For *h* = 1, one has

9 $$\frac{y\left(t\right)-y_{0}}{y_{\infty }-y_{0}}=X\left(t\right)=\frac{{\left(\frac{t}{\tau }\right)}^{a}}{{1+\left(\frac{t}{\tau }\right)}^{a}}$$

Equation 9 succeeded in fitting data from a broad spectrum of HTC reactions^42^ and, more interestingly, it mirrors the functionality of the Sips (Langmuir–Freundlich) adsorption isotherm, recently invoked successfully for modeling protein adsorption on activated HC.^43^ Adsorption isotherms like the Sips could arise in the presence of active surfaces with a fractal geometry. This latter finding strengthens this study’s hypothesis and encourages adopting fractal analysis in more in-depth investigations on HC properties. Finally, the fractal origin of eq 8 is demonstrable.^39^ The equation is formally identical to the solution of the fractal differential equations:

10 $$\frac{d^{\alpha }y\left(t\right)}{dt^{a}}=-\frac{1}{\tau }y{\left(t\right)}^{h}$$

The detailed mathematical procedure that leads to the solution is too long and out of the scope of this contribution. Nevertheless, some brief methodological hints could help the interested reader. In a nutshell, the procedure uses the Riemann–Liouville fractional derivative:

11 $$\frac{d^{\alpha }y\left(t\right)}{dt^{a}}=-\frac{1}{\Gamma \left(1-\alpha \right)}\frac{d}{dt}\int_{0}^{t}{\left(t-\tau \right)}^{-\alpha }y\left(\tau \right)d\tau$$

where Γ­(1 – α) denotes the γ function.

The solution of eq 10, with the initial condition *y*(0) *= y*_0_, connects to that of the simple first-order differential equation:

12 $$\frac{dy\left(t\right)}{dt}=-R\left(t\right)y\left(t\right)$$

Following the reliability theory, in the last equation, *R*(*t*) represents a time-dependent hazard function that refers to the already defined parameters:

13 $$R\left(t\right)=\frac{1}{t}\frac{{\left(\frac{t}{\tau }\right)}^{a-1}}{\left[1+\left(n-1\right)\left(\frac{t}{\tau }\right)\right]}$$

## 3 Materials and Methods

### 3.1 Materials

All of the reagents and chemicals were pure-grade products from the laboratory commodities market. The raw biomasses come from the district and are typical residues and wastes from local grooves and industries: FIR is widely available from carpentry of routine forest maintenance and POTATO and CARROT are massive production scraps of agro-food industries. The raw materials were oven-dried (POTATO and CARROT, 60 °C, 48 h; FIR, 105 °C, 24 h) and milled to 0.5 mm mesh size.

All experiments and analyses used ultrapure demineralized water.

### 3.2 HTC Reactions

Details on the custom-made stainless-steel 200 cm^3^ batch reactor and the service equipment are available elsewhere.^38^ The reactor loading occurred with ten grams of dry solid and 70 grams of water, after which the vessel was sealed and evacuated of air with a vacuum pump (ABM model 3EKF56). The warmup procedure lasted 20 min, with a 9 °C/min ramp. Reactions ran at 200 °C and six residence times (0, 10, 15, 30, 60, and 120 min). The residence time of 0 stays for a quenching immediately after the set point temperature is established. The cooling down procedure (compressed air direct blowing followed by cold water bath immersion) took 6 min, after which the products were recovered immediately. The gas phase (on average, 2% w/w of the dry biomass) was discharged. The separation of condensed products by filtration gave the liquid, stored at 4 °C, and the solid, oven-dried at 105 °C, up to constant weight and stored at room temperature. All kinetic runs were conducted once, provided that the overall relative error is about 4% in hundreds of previous experiments.

### 3.3 Analytical

HC ultimate compositions were determined with a PerkinElmer 2400 Series II elemental analyzer, according to the standard procedure ASTM D3176-89, 2002. Solid yields and dry weight determination went according to UNI EN ISO 18134-2, 2015.

The nitrogen sorption/desorption isotherms of biomass and hydrochars were measured with a high-speed surface area and pore size analyzer NOVA 1200e Alfates Quartachrome. The standard procedure allows for determining surface area, pore volume, pore size distribution, and the average pore diameter. Each sample was outgassed for 3 h at 100 °C before undergoing the adsorption/desorption cycle. Each of the previously described analyses was in triplicate.

## 4 Results and Discussion

### 4.1 Analysis of Model Equation Dynamics

Candidate equations should pass the test of fitting data. This prerequisite screening does not guarantee identifying proper models *per se* but is a vital clue for addressing more in-depth analyses. Models capable of explaining HTC data well crowd the literature. Searching for mechanistic explanations, model screening is laborious. However, the introductory nature of this contribution makes supernumerary examples unnecessary. Table 1 lists the selection of test models and their fractal-like versions in terms of the reaction time course. The equations are adaptations of those used in the adsorption kinetic studies referenced in the last column. Equations 2, 4, and 7, coming from the stochastic approach described in the Modeling section, complete the set to test against experimental data. First- and second-order kinetics, the most invoked model for describing mass-action mechanisms, fail at describing adsorption data in many cases. Therefore, specialists introduced the intermediate mixed-order concept,^44^ appearing in Table 1 as eq 18. The mixed-order mechanism, a linear combination of first- (eq 14) and second-order (eq 16), contains the additional parameter A: varying from 0 to 1, it weighs the contribution of the second-order kinetic to the overall rate.

**Table 1:** Selection of Candidate Equations in Normal and Fractal-Like (f) Forms

	kinetic model	time-course equation	parameters	references
T1	first-order	T1 X(t)=1−exp(−kt)	*k*	^44^
T1f	f-first-order	T1f $X\left(t\right)=1-\exp\left(-\frac{k}{h}t^{h}\right)$	*k*, *h*	^29^
T2	second-order	T2 $X\left(t\right)=\frac{\left(y_{\infty }-y_{0}\right)\mathrm{kt}}{1+\left(y_{\infty }-y_{0}\right)\mathrm{kt}}$	*k*	^44^
T2f	f-second-order	T2f $X\left(t\right)=\frac{(y_{\infty }-y_{0})\frac{k}{h}t^{h}}{1+\left(y_{\infty }-y_{0}\right)\frac{k}{h}t^{h}}$	*k*, *h*	^29^
T3	mixed-order	T3 $X\left(t\right)=\frac{1-\exp\left(-\mathrm{kt}\right)}{1-A\;\exp\left(-\mathrm{kt}\right)}$	*k*, *A*	^44^
T3f	f-mixed-order	T3f $X\left(t\right)=\frac{1-\exp\left(-\frac{k}{h}t^{h}\right)}{1-A\;\exp\left(-\frac{k}{h}t^{h}\right)}$	*k*, *h*, *A*	^29^

Figures 1–3 offer a bird’s eye view of the dynamics underlying the model equations. They report the advancement (*X*) as a function of a proper dimensionless time (θ), i.e., the abscissa is *t* divided by the combination of parameters specific to a particular model that gives a characteristic time. The curves are generated as functions of θ^*h*^, and the discussion deals with the effect of *h* on the dynamics featured by each equation. This artifice allows a comprehensive comparison between the models. According to the invoked autocatalysis, candidate equations should preferably generate S-shaped curves. eqs 14–18 do not exhibit the required behavior, regardless of the value of the parameters. This feature does not imply complete inappropriateness: data of short and long HTC reaction time tails should align along concave and convex curves, respectively, with no inflection points. Besides, some biomasses undergo an initial transformation at a temperature below that of the set point, i.e., during the batch reactor warmup transient, making the rate acceleration unobservable. In this last case, the entire set of experimental data lies within the long-time tail. Figure 1 shows the behavior of eqs TT1f and TT2f (parts a and b, respectively). Dashed lines trace the mother nonfractal equations (*h* = 1), and solid lines the corresponding fractal modifications. The eq TT1f family displays a faster kinetics than that of eq TT2f. To point out this, Figure 1 also shows the *X* = 0.9 threshold (90% of the phenomenon accomplished). Equation TT1f crosses the line at θ = 2.3, eq TT2f at 9. This feature also appears for the fractal modifications. For example, taking the fractality parameter at *h* = 3, eq TT1f crosses at θ = 1.90.3, eq TT2f at θ = 4.76. The family of eq TT3f depends on an additional parameter, A, making the graphical representation more complicated.

**Figure 1:**
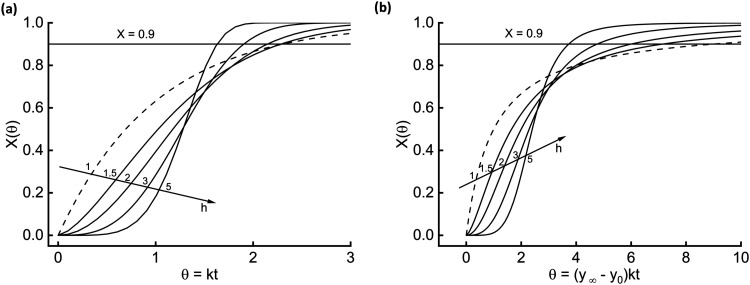
Dynamics associated with nonfractal and fractal eq TT1f (a) and eq TT2f (b).

To avoid excessive crowding while preserving the essence of dynamics, Figure 2a reports the effect of *h* while keeping the other parameter *A* fixed at 0.2, along with an additional curve for *A* = 0.8 and *h* = 2. The comparison of this last curve with the corresponding one at *A* = 0.2 highlights the high sensitivity toward the parameters.

**Figure 2:**
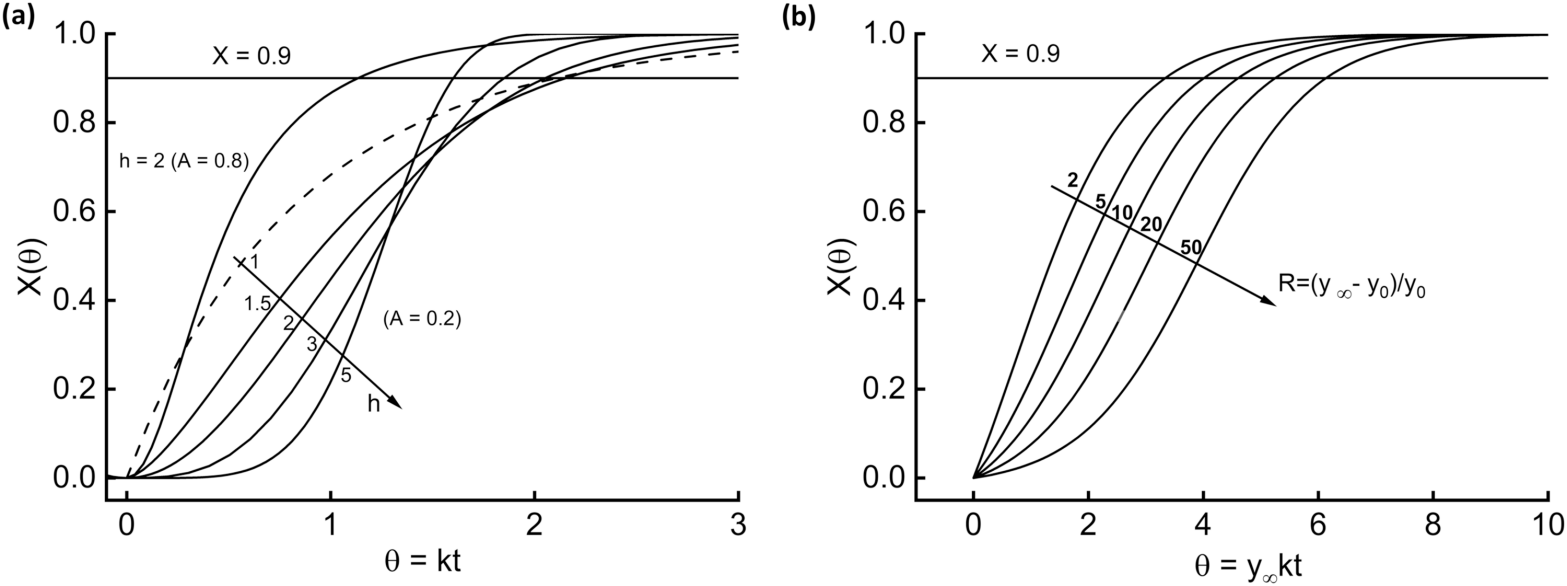
Dynamics associated with eq TT3f (a) and eq 2 (b).

The above analysis states that the fractalized eqs TT1f–TT3f produce S-shaped curves, and this finding makes them suitable for modeling HTC kinetics.

Equations 2, 4, and 7 belong to another approach toward fractal modeling, i.e., arise from mechanistic bases. Hence, they intrinsically generate the required dynamics.

The comparison of these further models with the previous ones requires the associated reaction advancement formulas. eq 8 is the appropriate one for the kinetic eq 7. Table 2 lists those for the remaining two, alongside other remarkable properties of all of the models useful for industrial process optimization. Particularly, for each model equation, Table 2 reports the rate at which the HTC proceeds, the time of inflection point appearance, and the time for bringing the process halfway to completion. These entries bring valuable information for appropriately sizing the HTC reactor and setting correctly the operational parameter.

**Table 2:** List of Significant Properties Associated with Model Equations

equation	*X* (*t*)	*t*_1/2_ (*y*)	*t*_1/2_ (*X*)	${\left[\frac{\mathrm{dX}}{\mathrm{dt}}\right]}_{t=0}$	$\max\left(\frac{\mathrm{dX}}{\mathrm{dt}}\right)$
	reaction advancement	time for *y* halfway between *y*_0_ and *y*_∞_	time for 50% advancement	initial reaction rate	time for the maximum reaction rate
2	$\frac{1-e^{-y_{\infty }\mathrm{kt}}}{1+\frac{y_{\infty }-y_{0}}{y_{0}}e^{-y_{\infty }\mathrm{kt}}}$	$\frac{1}{y_{\infty }k}\ln\left(\frac{y_{\infty }-y_{0}}{y_{0}}\right)$	$\frac{1}{y_{\infty }k}\ln\left(\frac{y_{\infty }+y_{0}}{y_{0}}\right)$	*y*_0_ *k*	$t=\frac{1}{y_{\infty }k}\ln\left(\frac{y_{\infty }-y_{0}}{y_{0}}\right)$
4	$\frac{1-e^{-y_{\infty }k/ht^{h}}}{1+\frac{y_{\infty }-y_{0}}{y_{0}}e^{-y_{\infty }k/ht^{h}}}$	$\sqrt[{h}]{\frac{h}{y_{\infty }k}\ln\left(\frac{y_{\infty }-y_{0}}{y_{0}}\right)}$	$\sqrt[{h}]{\frac{h}{y_{\infty }k}\ln\left(\frac{y_{\infty }+y_{0}}{y_{0}}\right)}$	0	≈
8	$\frac{{\left(1+h{\left(\frac{t}{\tau }\right)}^{a}\right)}^{1/h}-1}{{\left(1+h{\left(\frac{t}{\tau }\right)}^{a}\right)}^{1/h}}$	$\tau \sqrt[{a}]{\frac{{\left(2\frac{y_{\infty }-y_{0}}{y_{\infty }}\right)}^{h}-1}{h}}$	$\tau \sqrt[{a}]{\frac{2^{h}-1}{h}}$	0	$t=\tau \left(\frac{a-1}{a+h}\right)$
T1	1 – e^–*kt*^	$\frac{1}{k}\ln\left(2\frac{y_{\infty }-y_{0}}{y_{\infty }}\right)$	$\frac{1}{k}\ln\left(2\right)$	*k*	*t =* 0
T1f	$1-e^{-k/ht^{h}}$	$\sqrt[{h}]{\frac{h}{k}\ln\left(2\frac{y_{\infty }-y_{0}}{y_{\infty }}\right)}$	$\sqrt[{h}]{\frac{h}{k}\ln\left(2\right)}$	0	$t=\sqrt[{h}]{\frac{h}{k}}$
T2	$\frac{\left(y_{\infty }-y_{0}\right)\mathrm{kt}}{1+\left(y_{\infty }-y_{0}\right)\mathrm{kt}}$	$\frac{1}{y_{\infty }k}\frac{{y_{\infty }-2y}_{0}}{y_{\infty }-y_{0}}$	$\frac{1}{k}\frac{1}{\left(y_{\infty }-y_{0}\right)}$	*k*(*y*_∞_ – *y*_0_)	*t* = 0
T2f	$\frac{(y_{\infty }-y_{0})\frac{k}{h}t^{h}}{1+\left(y_{\infty }-y_{0}\right)\frac{k}{h}t^{h}}$	$\sqrt[{h}]{\frac{h}{k}\frac{y_{\infty }-{2y}_{0}}{y_{\infty }\left(y_{\infty }-y_{0}\right)}}$	$\sqrt[{h}]{\frac{h}{\left(y_{\infty }-y_{0}\right)k}}$	0	$t=\sqrt[{h}]{\frac{1}{k\left(y_{\infty }-y_{0}\right)}\frac{h^{2}-h}{h+1}}$
T3	$\frac{1-e^{-\mathrm{kt}}}{1-{\mathrm{Ae}}^{-\mathrm{kt}}}$	$\frac{1}{k}\ln\left(A+2\frac{y_{\infty }-y_{0}}{y_{\infty }}\right)$	$\frac{1}{k}\ln\left(2+A\right)$	$\frac{k}{{\left(A-1\right)}^{2}}$	*t* = 0
T3f	$\frac{1-e^{-k/ht^{h}}}{1-{\mathrm{Ae}}^{-k/ht^{h}}}$	$\sqrt[{h}]{\frac{h}{k}\ln\left(2-A\right)}$	$\sqrt[{h}]{\frac{h}{k}\ln\left(2-A\right)}$	0	≈

Figure 2b shows the dynamics of model eq 2. The higher the relative span between the end-point and initial value (parameter R), the more accentuated the S-shape and the lower the reaction rate. Equation 4 is the fractal version of eq 2 and, as such, contains the parameter *h*, which modulates the degree of fractality.

In Figure 3a, the circles encompass three couples of curves, obtained for different values of the parameter R. Solid lines refer to *h* = 2 and dashed lines to *h* = 1 for reference (no fractality, eq 2 holds). Fractality steepens the sigmoidal curves. Finally, eq 8 is the more complete kinetic model this study contemplates. There is an additional parameter, *a*, which accounts for the time-variability of the lumped kinetic constant. Figure 3b offers a comprehensive glance at the reaction advancement time course. Circles locate two triplets of curves at constant *h*, and arrows indicate increasing *a*. Curves with *a* = 1 (dashed lines) do not display an inflection point since it would appear for a negative time.

**Figure 3:**
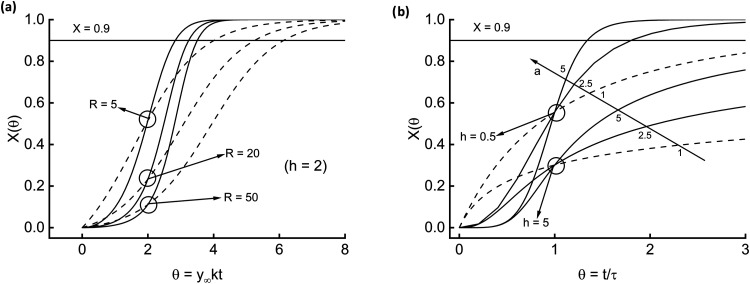
Dynamics associated with eq 4 (a) and eq 8 (b).

The above discussion clarifies fractal kinetics’ potential role as a further tool for HTC modeling. All equations in Table 2 contain a few parameters, overlook the awkwardness of the mass-action approach, and are straightforwardly insertable into mass balance over HTC reactors. These advantages make the introduction of fractal analysis not academic speculation but a valiant support for chemical engineers who can afford the industrial process of biomass hydrothermal carbonization.

### 4.2 Estimation of Sample Fractality

Several techniques allow measuring surface fractal dimension, such as scanning electron microscopy, X-ray computer tomography, gas adsorption methods, mercury intrusion porosimetry, and image processing.^45^A complete validation of HTC process fractality and how this reflects in each of the proposed equations would require future, in-depth studies. The scope of this paper is to furnish sufficient hints to make the prosecution of the investigation worth affording.

Low-temperature nitrogen adsorption/desorption is the proper recipe for sounding out quickly the surface complexity of meso- and nanopores in carbonaceous materials.^46^ The analysis of this study concerns FIR, CARROT, and POTATO, with significantly different average lignin percentage content (30, 8, and 1.5, respectively).^47–49^ Figure 4 refers to FIR, an example of the woody material. Part a) shows the isotherms and underlines a significant desorption hysteresis, which could further signal complexity. The amount of gas adsorbed is relatively tiny compared to other materials, as expected for a native, nonactivated solid. The question arises if and to what extent the HTC process could alter the geometry of the matrix. Part (b) furnishes initial evidence, showing that the HC recovered after 120 min of reaction behaves differently from the parent biomass. Nitrogen uptake augments, especially in the high relative pressure range (smallest size pores); the effect of hysteresis lessens. Biomass refining and carbon redeposition, which rule the dynamics of primary and secondary HC formation, also affect the geometry of the carbonaceous material.

**Figure 4:**
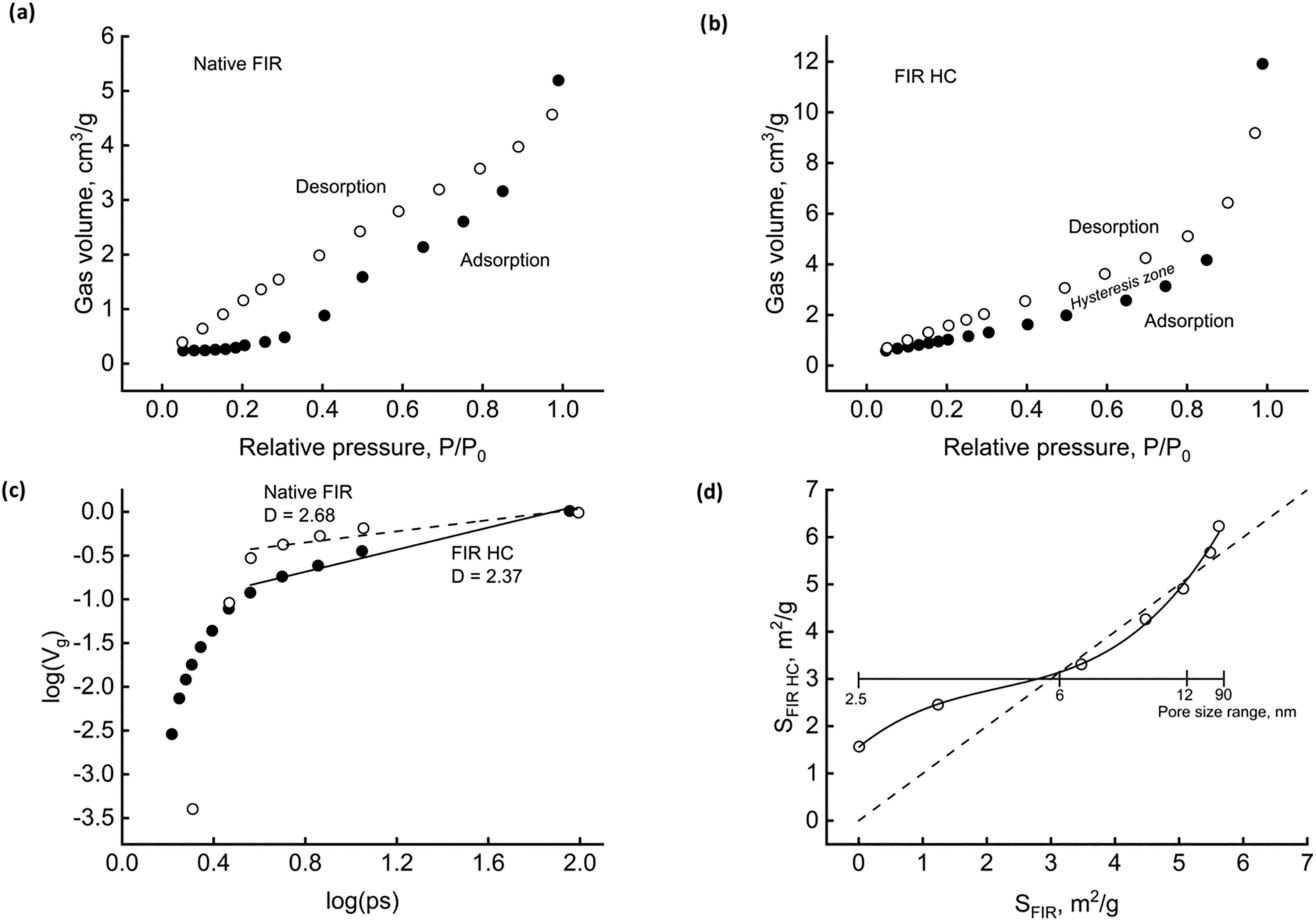
FIR surface characterization. Adsorption/desorption isotherms for biomass (a) and HC (b). Fractal dimensions (c). Pore size variation during HTC (d).

Part c illustrates one of the more usual methods for quickly estimating D, the pore surface fractal dimension, from adsorption data. The logarithmic plot shows the cumulative pore fraction volume (*V*_g_) as a function of pore size (ps). Wherever data align along a straight line, the slope represents (3D).^45^ This circumstance occurs in the meso- to macropore size region for both native and hydrothermally treated FIR. HC fractality reduces from 2.68 (native biomass) to 2.37 for the HC. Provided that surface complexity increases as *D* increases from 2 to 3, the matrix structure highlights fractality, thus confirming that scale-invariance processes rule the time evolution of biomass hydrothermal carbonization.

Part (d) points out that the HTC process alters the intimate structure of the solid phase, reporting the HC pore surface as a function of the native biomass one. Each point refers to a pore size range (an auxiliary scale helps read the measures). A full line connects data to help read the trend. The dashed line is the bisector: the points above show that additional surfaces become available during the treatment, while the points below signal pore deconstruction. HTC has a favorable effect on nano- and macroporosity and tends to reduce mesopores.

Figures 5 and 6 refer to the two low-lignin biomasses, POTATO and CARROT. The adsorption/desorption patterns of Figure 5a demonstrate that the native CARROT behaves similarly to the woody material, except for the predictable lesser gas uptake capacity. Two hours of HTC treatment cancel the hysteresis and increase the material’s gas capacity (Figure 5b). The buildup of fractality resulting from HC formation appears dramatically in Figure 5c. While no evident fractality is detectable on the native biomass, CARROT HC shows it to a certain extent (*D* = 2.31). Finally, Figure 5d shows the effect of HTC on the surface available for nitrogen adsorption. Whatever the pore size range, the specific surface area expands significantly compared to that of the parent biomass. All data points place themselves above and far from the bisector line.

**Figure 5:**
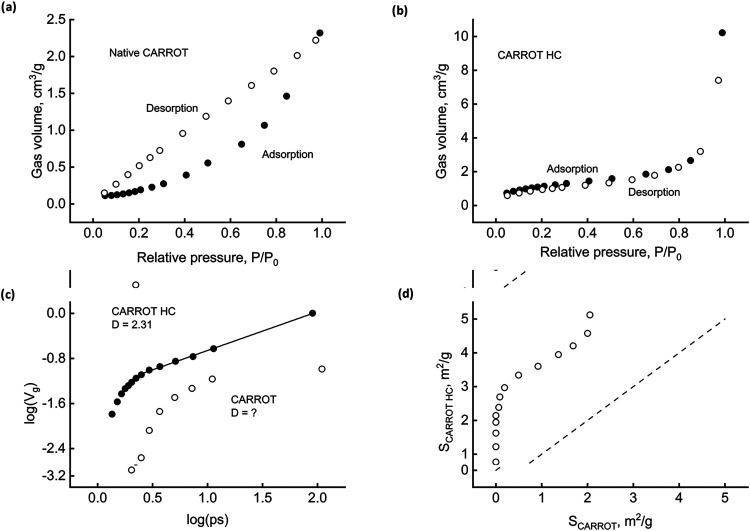
CARROT surface characterization. Adsorption/desorption isotherms for biomass (a) and HC (b). Fractal dimensions (c). Pore size variation during HTC (d).

**Figure 6:**
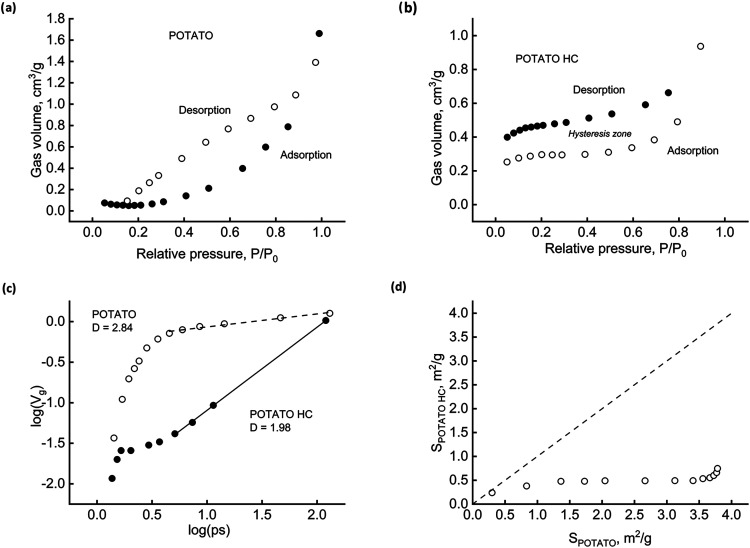
POTATO surface characterization. Adsorption/desorption isotherms for biomass (a) and HC (b). Fractal dimensions (c). Pore size variation during HTC (d).

Data referring to POTATO (Figure 6) are even more interesting and deserving of further, advanced investigation. Part (a) exhibits the lowest gas uptake and differently closed hysteresis cycle of this low-lignin native biomass. Part (b) depicts two noticeable findings: the desorption is now not wholly reversible (open hysteresis loop); the HC performs worse than the parent biomass, halving the nitrogen adsorption capacity. Part (c) reports further unexpected results on fractality estimation in that the native biomass features a high index (*D* = 2.84). At the same time, the corresponding HC has none and, in addition, a peculiar cumulative fraction volume trend. Part (d) further highlights the difference between POTATO and the other biomasses. Specific surface variation with respect to the native material is countertrend: the HTC process reduces the surface available for gas–solid interactions in all of the pore size ranges.

This section’s results state the strong connection between surface fractal organization and the hydrothermal carbonization reactions, which transform native lignocellulosic materials into HC. Table 3 gathers the main parameters proving this evolution. One can observe the variation of estimated *D* parameters. Further evidence is that HTC enlarges pore volume up to four times, alters pore size distribution, as the average values demonstrate, and affects the specific surface of medium- and high-lignin containing materials. Because of the small initial amount of the insoluble matrix, POTATO entrusts the HC growth almost entirely to the secondary HTC reactions, which are responsible for depositing carbonaceous materials from polycondensation reactions. Accordingly, a more in-depth investigation of secondary HC formation kinetics is required. Further investigation will clarify whether fractal formalism is appropriate only for heterogeneous, lignocellulosic solids or can extend to homogeneous systems (e.g., fructose or starch solutions). In other words, if the fitting successes for low-lignin materials are purely empirical or a consequence of fundamental mechanistic conditions.

**Table 3:** Synopsis of Hydrochars’ Pore Properties

	surface area (m^2^/g)	average pore size (nm)	volume (cm^3^/g)	fractal dimension
	ADS/DES	ADS/DES	ADS/DES	
FIR	5.62/10.22	3.65/19.5	0.010/0.010	2.68
HC	6.23/12.04	2.92/1.96	0.019/0.021	2.37
CARROT	2.05/5.04	3.66/1.95	0.004/0.005	ND
HC	5.12/4.60	1.35/1.43	0.016/0.016	2.31
POTATO	1.04/3.78	5.11/1.95	0.003/0.004	2.84
HC	0.966/0.725	1.37/1.42	0.007/0.008	1.98

### 4.3 Assessment of Model Equations

A heuristic approach for sifting through models is trimming unnecessary complexity and selecting the simpler one that can predict the reaction’s dynamics satisfactorily. Accordingly, the final part of this study deals with testing the models against HTC experimental data. The ideal data set should span 2–3 h of reaction with at least a dozen equally spaced time points coming from raw materials of different textures and lignin content. Data harvesting is problematic since only a few literature studies have thoroughly analyzed the reaction time course. Additionally, on-purpose HTC reactions, performed according to the description of the experimental section, furnished Supporting Information. Table S1 appears in the Supporting Information file as a synopsis of all of the data employed with references to their sources. These recent anthological examples encompass a sufficiently wide range of biomass typologies, reaction conditions, duration, and modeling approaches to represent a stress test on the fractal equations. The analysis deals with cases of satisfactory convergence (Levenberg–Marquardt algorithm) and discards all equation data set combinations that mismatch this criterion. The compendium of the obtained *R* ^2^-values appears in Supporting Information, Table S2, which reports a range from 0.91202 to 0.99998, with a mean value of 0.97957. In most cases, fractal equations fit literature data better than original models. Figures 7 and 8 report the parity plots (predicted vs observed data).

**Figure 7:**
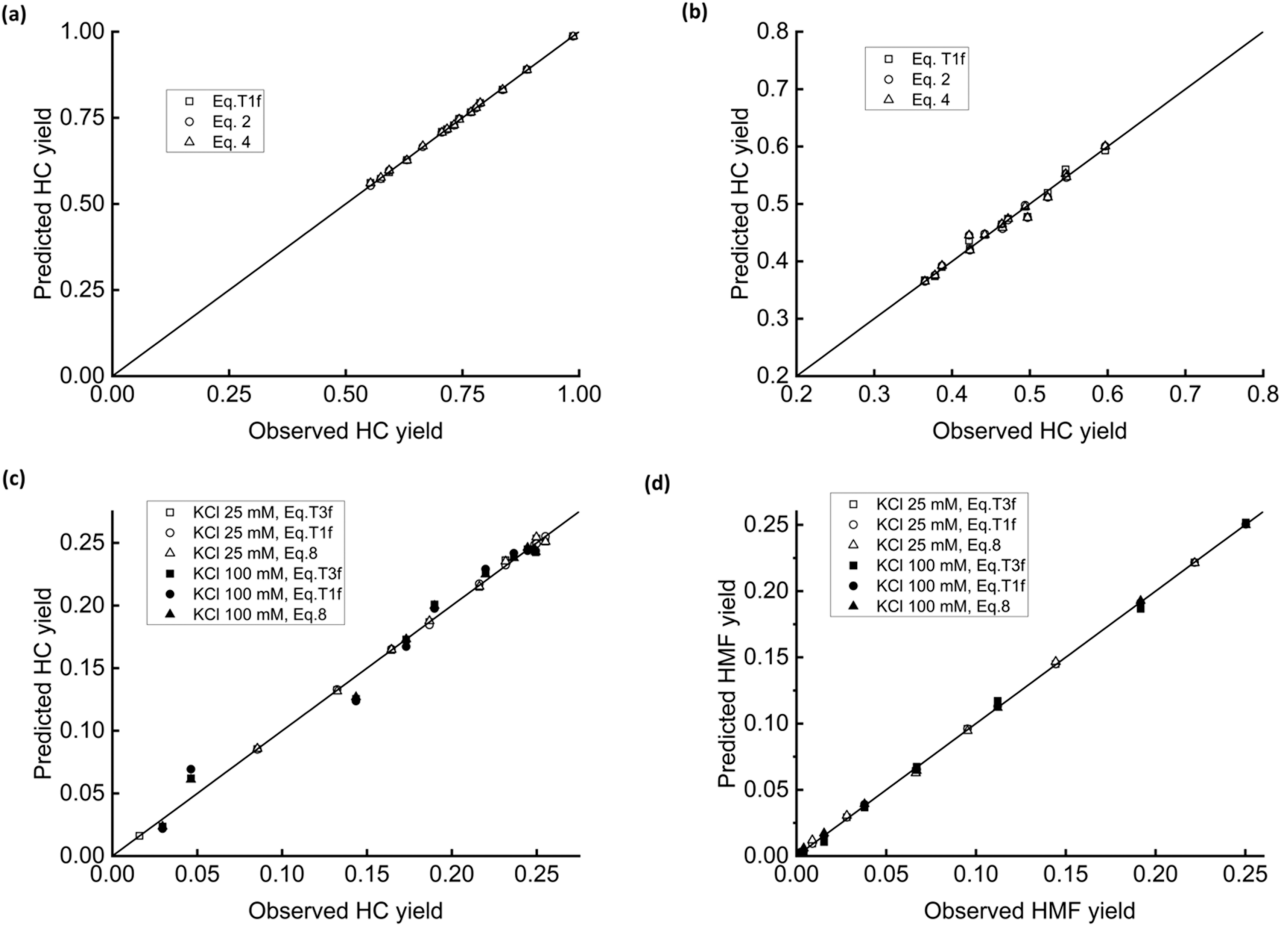
Fractal fittings. HC yield, ref 50, pine (a); HC yield, ref 50, rapeseed (b); HC yield, ref 51, fructose (c); and HMF concentration, ref 51, fructose (d).

Parts (7a) and (7b) refer to HC yield from pine and rapeseed raw biomasses, respectively. The authors^50^ set up a compartmental scheme based on first-order lumped reactions. Before performing experiments independently, they tested the model against a substantial and broad range of literature data and obtained a satisfactory agreement between observed and predicted values. The model’s reliability was poorer against their on-purpose experiments, with deviations as high as 4%. The results of fractal analysis on data regarding pine biomass (Figure 7, part a) are encouraging. The three best-performing equations yield points almost perfectly aligned along the bisector line, with identical regression coefficients of 0.99998. A simple, two-parameter fractal first-order reaction, eq 2, describes data successfully with mathematical parsimony. Although placed close to the bisector line, the points representative of rapeseed display a certain scattering in Figure 7b. Regression coefficients are set to 0.9996 using the same equations as part (a). A possible explanation is that rapeseed contains less lignin and cellulose. This result hints at the importance of lignin as a fractal scaffold in the materials undergoing HTC.

Figure 7c,d concern a completely different experimental structure, wherein the parent material is pure carbohydrate, fructose, the HTC makes use of a catalyst (KCl 25 and 100 mM), and time-monitoring also examines a liquid-phase product, 5-hydroxymethylfurfural (HMF). The reference authors^51^ adopted a 10-step mass-action mechanism, which is in excellent conformity with their data. The fractal analysis shows that eqs 8, TT3f, and TT1f give the best results. Part c) depicts the fittings on the HC yield. Regression coefficients range from 0.96889 to 0.99952 and, generally, eq 8 performs better than the fractal-like first- and mixed-order models. Interestingly, the higher the catalyst concentration, the higher the data scattering. Future experiments should assess the reliability of the fractal description of catalyzed HTC of homogeneous solutions. Figure 7d shows that the prediction of HMF yield is excellent (*R* ^2^ ≥ 0.999). In this latter case, catalyst concentration appears irrelevant, suggesting that the catalyst interferes only with the solid-phase texture.

Figure 8a reports on a different raw material, waste from the slaughterhouse industry. In the original paper, a four-step compartmental network matched kinetic data at three reaction temperatures (160, 200, 240 °C).^52^ Fractal equation data reprocessing used eqs TT1f, 4, and 8. Regression coefficients range around 0.92 for the lower and higher temperatures and around 0.98 for the intermediate temperature. The fitting, still satisfactory, is less good than those obtained with other substrates. A possible cause would be the waste’s relatively high nitrogen content, which triggers side reactions that are not well-modeled by the fractal approach. Part b) of Figure 8 shows how the fractal analysis behaves at short HTC times (up to 6.5 min) with a woody raw material. The authors^24^ tested the reliability of using a continuous distribution of activation energies, intentionally keeping the reactor under a thermal transient. eqs TT1f, TT3f, and 8 result the best-performing ones (0.962 < *R* ^2^ < 0.962). Fractality also steers the reactions concurrent with the warm-up.

**Figure 8:**
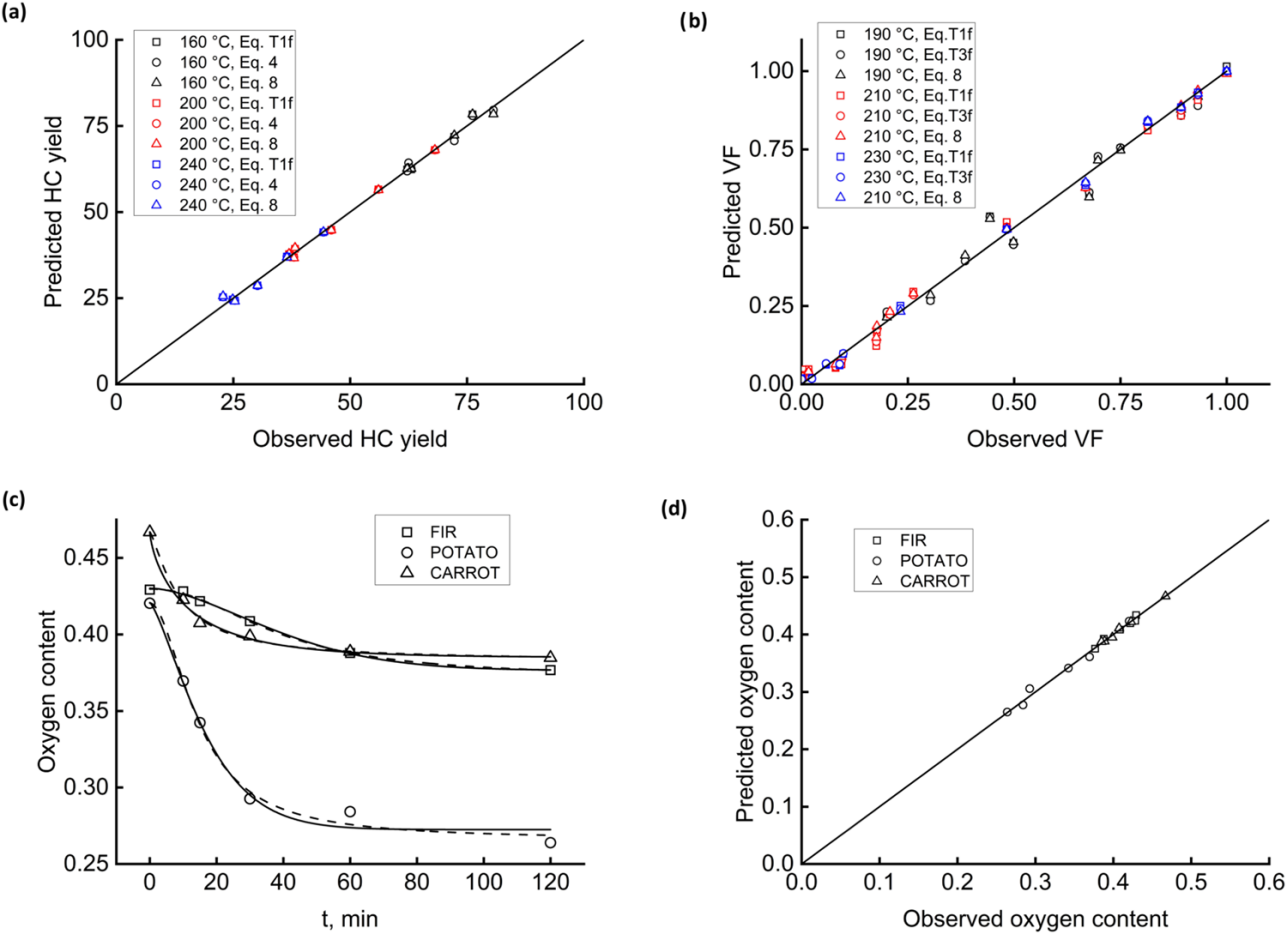
Fractal fittings. HC yield, ref 52, paunch waste (a); volatile fraction, ref 24, woody scraps (b); HC oxygen content, this study, FIR, POTATO, and CARROT: eq 8, red line, and eq TT1f, black line (c); and parity plot for eq 2 (d).

The last part of this discussion concerns data produced by experiments expressly done with the three native materials, FIR, POTATO, and CARROT, whose composition in lignin, cellulose, and hemicellulose varies significantly. The target analyte is the oxygen content, which probes whether the proposed model equations also monitor the time course of HC composition.

Figure 8c compares the fitting of eq 8, red lines, and TT1f, black lines. For the high-lignin material FIR, the two fitting lines are indistinguishable from each other (average *R* ^2^ = 0.995). The intermediate lignin content of CARROT gives a slight divergence at a short reaction time, which progressively vanishes as HTC proceeds. Regression quality is still satisfactory (average *R* ^2^ = 0.99). POTATO, a low-lignin, prevalently starchy material, should produce HC mainly through the secondary pathways. The corresponding fitting lines differ from each other in the entire range of reaction times and give the worst average *R* ^2^ (0.97). A cross-check with Table 3 highlights that HTC dismantles fractality. The parent material possesses fractality (*D* = 2.84), and the ultimate HC does not (*D* = 1.98). Fractal equations remain relatively reliable in this case because the sparse lignin drives the deconstruction reaction within the constraints of fractality.

Figure 8d completes the fitting tests on the three materials, showing the parity plot of eq 2. Data, although relatively well aligned along the bisector line, hints at the incompleteness of this equation for explaining the starchy materials’ HTC dynamics.

As pointed out, available data sets suffer from a scarcity of time points. Despite a satisfactory *R* ^2^, this insufficiency leaves more complex equations vulnerable to overfitting. The precept of parsimony compels us to prefer equally well-fitting models with the minimum number of parameters. A tool for addressing the selection is Akaike’s information criterion^53^ (AIC), which quantifies the lost information using a specific model for describing data. The lesser the loss, the better the model. The AIC tests on all the fittings of Table S2 involved assessing 55 different pairs on the same data set. Thirty-seven combinations returned a preference; for the remaining 18, the information was insufficient for discrimination. The complete set of tests appears now in the Supporting Information as Table S3. The simpler model (TT1f) is neatly the best of TT3f and 4 but worse than the more complex model 8. Other entries of Table S3 confirm that Occam’s razor (avoid unnecessary complexity) works in most cases, but not as far as eq 8 is concerned. The demonstrable fractal origin of this last equation places it on a higher step than the other ones. However, the complete assessment of this four-parameter model would require more experiments to have a congruous number of time points available.

In the future, a more rigorous analysis would be imperative for assessing the ability of models to furnish reliable values for the parameters. In the frame of the present contribution, the interested reader can refer to the Supporting Information that reports the residual of all of the forty-five fits.

The above anthological discussion dealt with a broad range of raw biomasses, HTC conditions, modeling approaches, and properties monitored. The results encourage prosecuting research in many ways: measure accurately the HC fractal dimension during the HTC proceeding and search for correlations between fractal surface properties, kinetic behavior, and chemical composition analysis (e.g., lignin/cellulose composition or aromaticity). Finally, a comparison with the recently developed multiscale modeling approaches could be valuable for a combined description of these complex systems.

## 5 Conclusions

Fractality plays a role in the transformation of water-biomass slurries into hydrochar. The evidence suggests the usefulness of treating the HTC reacting environment as a complex system driven by microscale reactions constrained into fractal topological boundaries. Fractal kinetics deserves to flank the more traditional, bulk-phase-based models to contribute to the full advancement of knowledge of the hydrothermal process. The proposed panoply of equations gives satisfactory fitting results over a broad range of literature data. The parsimony in the number of parameters necessary for explaining the HTC time course is advantageous for the scale-up to industrial processes. The evidence of this contribution stimulates the prosecution of investigations and could intrigue researchers who specialize in surface studies and fractal modeling to make their peculiar contribution to the field.

## Supplementary Material



## Notes

Synopsis of data sources for fitting equations (Table S1); compendium of regression performances (Table S2); Akaike’s criterion outcomes (Table S3); summary of symbols (Table S4); mechanistic diagram of hydrochar formation within fractal constraints (Figure S1); and residual plots of the fittings (Figures S2/1–2) (PDF)
