# Supplementary material for: Fractal Kinetic Analysis of Biomass Hydrothermal Carbonization
Source: ACS Omega. 2025 Jul 30;10(31):34721–32. doi: 10.1021/acsomega.5c03676 (PMC12355234; doi:10.1021/acsomega.5c03676)
Supplement: Supplementary file 1 [file ao5c03676_si_001.pdf]

Supporting information

Fractal kinetic analysis of biomass hydrothermal carbonization

Alberto Gallifuoco*[1] and Luca Taglieri[1]

[1]University of L'Aquila - Department of Industrial and Information Engineering & Economics; Piazzale Ernesto Pontieri 1, Monteluco di Roio, 67100 - L'Aquila – ITALY

Table S1. Synopsis of data sources for fitting equations to experimental evidence

Table S2. Compendium of regression performances

Table S3. Akaike criterion on pairs of model equations

Table S4. Summary of symbols

Figure S1. Mechanistic diagram of hydrochar formation within fractal constrains

Figure S2/1. Residual plots of the fitting.

Figure S2/2. Residual plots of the fitting (continued).

* Corresponding author
e-mail: alberto.gallifuoco@univaq.it

Table S1. Sources of experimental HTC data for models' assessment

Substrate	Property	T °C	Duration min	Kinetic scheme	Steps	Ref.
Rapeseed, pine	HC yield	190-250	0-120	Compartmental	5	50
Fructose	HC yield	200	0-180	Mass-action network	10	51
Fructose	HMF yield, liquid	200	0-180	Mass-action network	10	51
Paunch waste	HC yield	160-240	0-240	Compartmental	4	52
Woody scraps	Volatiles	190-230	0-6.5	Continuous lumping	-	24
Fir	Oxygen content	200	0-120	Fractal, lumped	-	This study
Carrot scraps	Oxygen content	200	0-120	Fractal, lumped	-	This study
Potato scraps	Oxygen content	200	0-120	Fractal, lumped	-	This study

Table S2. Synopsis of HTC experimental data fittings

	Substrate	Property	(Equations) R^2			Ref.
			A	B	C	
1	Rapeseed	HC yield	(T1f) 0.99971	(2) 0.99957	(4) 0.99959	50
2	Pine	HC yield	(T1f) 0.99997	(2) 0.99998	(4) 0.99998	50
3	Fructose (KCl 25 mM)	HC yield	(8) 0.99783	(T3f) 0.99815	(T1f) 0.99826	51
4	Fructose (KCl 100 mM)	HC yield	(8) 0.99952	(T3f) 0.97460	(T1f) 0.96889	51
5	Fructose (KCl 25 mM)	HMF yield, liquid	(8) 0.99850	(T3f) 0.99967	(T1f) 0.99972	51
6	Fructose (KCl 100 mM)	HMF yield, liquid	(8) 0.99954	(T3f) 0.99987	(T1f) 0.99820	51
7	Paunch waste (160 °C)	HC yield	(T1f) 0.92580	(4) 0.91780	(8) 0.91912	52
8	Paunch waste (200 °C)	HC yield	(T1f) 0.98554	(4) 0.98385	(8) 0.97958	52
9	Paunch waste (240 °C	HC yield	(T1f) 0.92705	(4) 0.92401	(8) 0.91202	52
10	Woody scraps (190 °C)	Volatiles	(8) 0.96222	(T3f) 0.95443	(T1f) 0.96358	24
11	Woody scraps (210 °C)	Volatiles	(8) 0.99515	(T3f) 0.99443	(T1f) 0.99031	24
12	Woody scraps (230 °C)	Volatiles	(8) 0.97945	(T3f) 0.99759	(T1f) 0.99737	24
13	Fir	Oxygen content	(T1f) 0.99402	(2) 0.95466	(8) 0.99603	This study
14	Carrot scraps	Oxygen content	(T1f) 0.98752	(2) 0.98792	(8) 0.99107	This study
15	Potato scraps	Oxygen content	(T1f) 0.97331	(2) 0.97184	(8) 0.98365	This study

Table S3. Akaike criterion on pairs of model equations

Model	T1f	T3f	2	4	8
**T1f**	-	7+, 0-, 0=	0+, 0-, 9=	6+, 3-, 0=	2+, 8-, 3=
**T3f**	0+, 7-, 0=	-			0+, 7-, 0=
**2**	0+, 0-, 9=		-	0+, 6-, 0=	3+, 0-, 0=
**4**	3+, 6-, 0=		6+, 0-, 0=	-	0+, 2-, 1=
**8**	8+, 2-, 3=	7+, 0-, 0=	0+, 3-, 0=	2+, 0-, 1=	-

(n1+, n2-, n3=) means that comparing a model with any of the others, it performs better in n1 cases, worse in n2 cases, and indifferently in n3 cases.

Table S4. Summary of symbols

Symbol	Units	Significance
τ	s	Characteristic time
A	-	Mixed-order modulator
a	-	Rate constant modulator
h	Model-depending	Fractal-like constant
k	s^{-1}	First-order kinetic constant
$t_{½}(X)$	s	Halfway time for X
$t_{½}(y)$	s	Halfway time for y
X	-	Reaction advancement
y	Model-depending	Time-depending property
y_{∞}	Model-depending	End-point y value
y_0	Model-depending	Initial y value

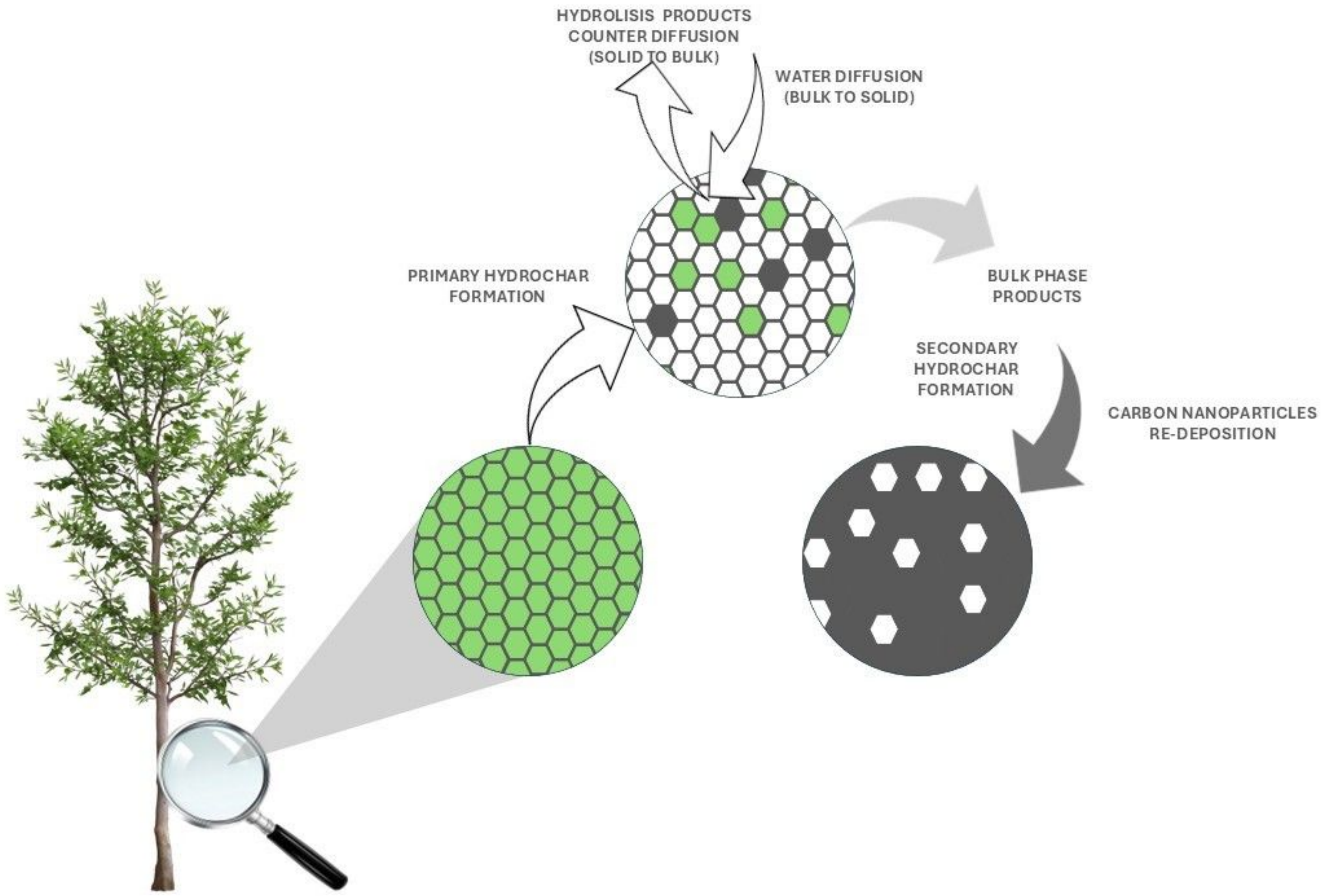

Figure S1. Mechanistic diagram of hydrochar formation within fractal constrains

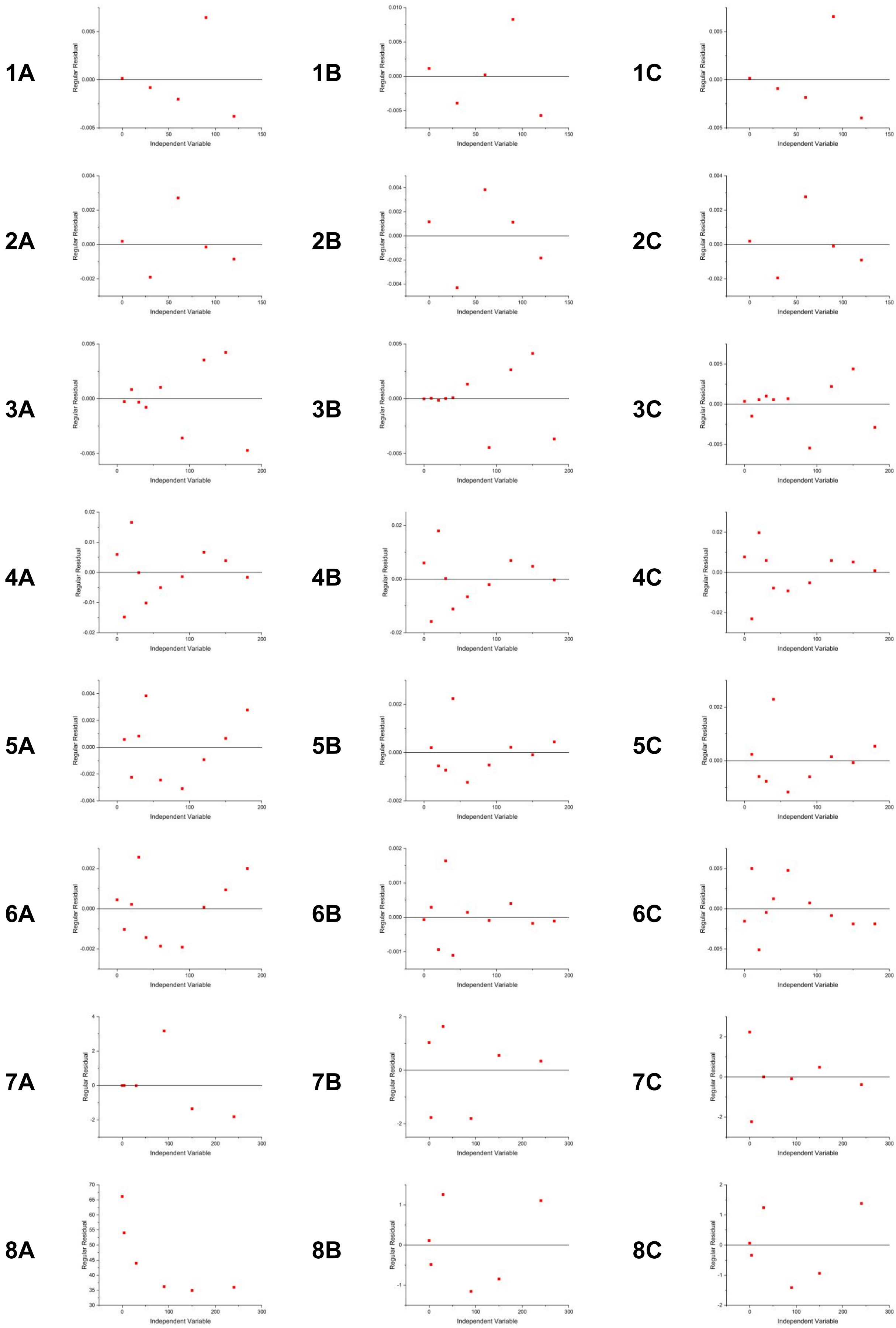

Figure S2/1. Residual plots of the fitting.

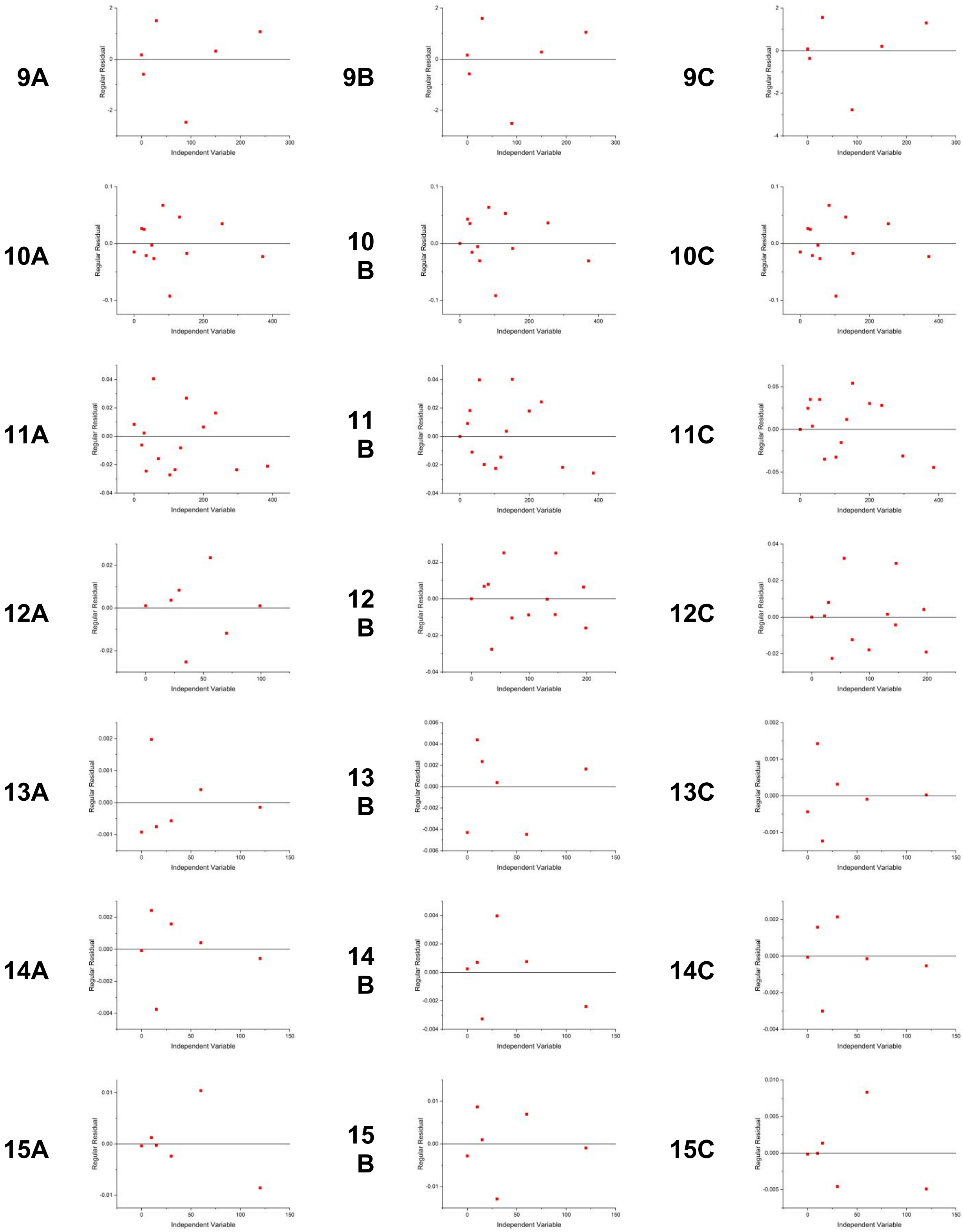

Figure S2/2. Residual plots of the fitting (continued).
